# Association between Serum Potassium with Risk of Onset and Visual Field Progression in Patients with Primary Angle Close Glaucoma: A Cross-Sectional and Prospective Cohort Study

**DOI:** 10.1155/2022/2275171

**Published:** 2022-06-23

**Authors:** Yichao Qiu, Jiaojiao Wei, Jian Yu, Yingzhu Li, Mingxi Shao, Jun Ren, Wenjun Cao, Shengjie Li, Xinghuai Sun

**Affiliations:** ^1^Clinical Laboratory, Eye & ENT Hospital, Fudan University, Shanghai 200031, China; ^2^Eye Institute and Department of Ophthalmology, Eye & ENT Hospital, Fudan University, Shanghai 200031, China; ^3^NHC Key Laboratory of Myopia (Fudan University), Key Laboratory of Myopia, Chinese Academy of Medical Sciences, Shanghai 200031, China; ^4^Shanghai Key Laboratory of Visual Impairment and Restoration, Shanghai 200031, China

## Abstract

Evidence suggests that ion metabolism may be associated with oxidative stress in the ocular tissue in glaucoma patients. This study is aimed at determining whether serum ion levels are associated with the onset and/or visual field (VF) progression of PACG. A total of 265 PACG and 166 healthy subjects were included in the cross-sectional study. Meanwhile, 265 subjects with PACG were followed up every six months for at least two years in the cohort study. All subjects were evaluated for serum concentrations of ions (calcium, phosphorus, potassium (K^+^), sodium, and chlorine) and underwent VF examination. Logistic regression analysis was performed to assess the risk factors for PACG. Cox regression analyses and Kaplan-Meier survival analyses were performed to identify factors associated with VF progression in PACG subjects. In the cross-sectional study, the K^+^ level (4.31 ± 0.39 mmol/L) was significantly higher in the PACG group than in the normal group (4.16 ± 0.35 mmol/L, *P* < 0.001). Multiple logistic regression showed that the increased K^+^ level was a risk factor of PACG (OR = 2.94, 95%CI = 1.63–5.32, *P* < 0.001). In the cohort study, there were 105 PACG subjects with progression and 160 PACG subjects without progression. The progression group had significantly higher baseline serum K^+^ levels (4.41 ± 0.37 mmol/L) than the no progression group (4.25 ± 0.39 mmol/L) (*P* = 0.002). The increased level of K^+^ at baseline was associated with faster VF progression (HR = 2.07, 95%CI = 1.23–3.46, *P* = 0.006). PACG subjects with higher baseline K^+^ levels had significantly lower VF nonprogression rates (51.94%) than subjects with lower K^+^ levels (68.38%, log-rank test *P* = 0.01). This study found that increased serum K^+^ level is a risk factor of PACG and is associated with faster VF progression in PACG, which might result from its influence on the oxidative stress process.

## 1. Introduction

Glaucoma is a neurodegenerative disease and a common cause of irreversible blindness which brings a heavy burden to society over the world [1]. The primary angle close glaucoma (PACG) is the main type of glaucoma in Asia, characterized by the closure of the anterior chamber angle [2]. Elevated intraocular pressure (IOP) is well accepted as the risk factor for PACG onset and development, lowering IOP is currently the only treatment that can slow the progression but not halt it [3]. To advance the prediction and prevention of PACG [4], other critical risk factors of glaucoma were proposed including genetic background [5, 6], vascular dysregulation [7], corneal thickness [8], personality, and stress [9].

Oxidative stress is one of the most recognized processes in the pathology of glaucoma [10, 11]. Oxidative stress might damage the trabecular meshwork resulting in the increase of IOP and might be harmful to retinal ganglion cells (RGCs) leading to glaucomatous visual defect [12–15]. Our previous studies also found that the serum levels of oxidative stress biomarkers were associated with the risk of PACG [16]. The ions maintain the balance of prooxidative and antioxidative processes in the ocular tissue of glaucoma patients [17]. However, whether the circulation ion levels were changed in PACG subjects remains uncertain.

We hypothesized that serum ion alterations are associated with the risk of PACG and the progression of VF loss. The serum levels of five ions, calcium, phosphorus, potassium (K^+^), sodium, and chlorine, were detected in PACG and normal subjects. Furthermore, the association between baseline serum ion levels and VF progression was assessed in PACG subjects.

## 2. Materials and Methods

This study was conducted at the Eye and Ear, Nose, and Throat (ENT) Hospital of Fudan University, Shanghai, China. The study obtained Institutional Review Board/Ethics Committee approval from the Ethics Committee of the Eye and ENT Hospital and was in accordance with the principles of the Declaration of Helsinki. Informed consent was obtained from all subjects.

### 2.1. Subject

In the cross-sectional study, PACG subjects diagnosed in the Department of Ophthalmology and Visual Sciences at the Eye and ENT Hospital of Fudan University were recruited from June 2016 to June 2018. The evaluation of serum ion level and ocular examination was performed the first time they came to the hospital. During the same period, age-matched normal subjects were recruited from the health-examined population and tested the serum ions.

In the cohort study, PACG subjects were recruited and followed up for at least two years in the Department of Ophthalmology and Visual Sciences at the Eye and ENT Hospital of Fudan University. The VF was detected at the first time they visited the hospital and 6, 12, 18, and 24 months after that.

### 2.2. Diagnostic and Inclusion Criteria

All PACG subjects were diagnosed by glaucoma specialists. PACG is defined as narrow anterior chamber angle, glaucomatous optic neuropathy, and typical and reliable VF defects, which were described in our previous studies [18–21]. During slit-lamp and gonioscopic examinations, the eye has ≥180° of the invisible posterior pigmented trabecular meshwork was determined as a narrow anterior chamber angle. The glaucomatous optic neuropathy was determined by notching or neuroretinal rim thinning with a vertical cup-to-disc ratio (VCDR) of >0.7 attributable to glaucoma. The glaucomatous VF defects were defined as having ≥3 contiguous non-edge contiguous points in the same hemifield with a possibility <5% of being present in normal eyes (one of which was <1%). The reliability criteria include fixation loss rate < 20%, false positive rate < 15%, and false negative rate < 15%. All PACG subjects received the topical IOP-lowing medication and had laser iridotomy over two months ago, but no ocular operational treatment within the previous 2 months and during the follow-up period. Normal control subjects with open anterior chamber angle, absence of glaucomatous symptoms, and family history were enrolled in the study.

All PACG and normal subjects met the inclusion criteria as follows: no secondary glaucoma or any other eye disease with visual acuity, or VF deficiency (including but not limited to cataract, age-related macular degeneration, and diabetes retinopathy); no surgery within the previous 2 months; no systemic diseases including autoimmune disease, metabolic syndrome, acute infectious disease, or cancer; and not taking drugs that could affect the serum ion levels.

**Figure 1:**
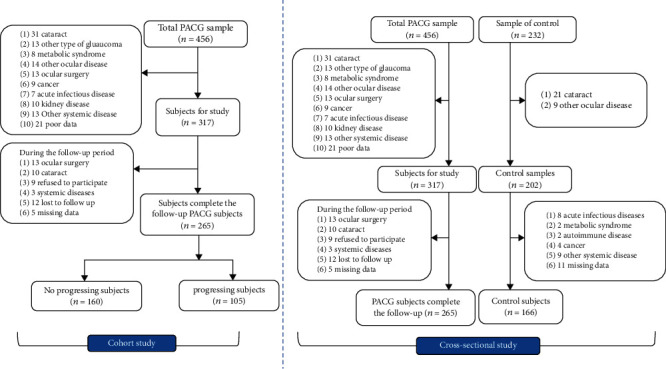
The flow chart of the cross-sectional and cohort study. PACG: primary angle close glaucoma.

In the cross-sectional study, 191 PACG subjects and 66 normal subjects were excluded based on the inclusion criteria. Finally, 265 PACG subjects and 166 normal subjects were enrolled. In the cohort study, 139 subjects were excluded according to the inclusion criteria, and 52 subjects were not fulfilled the follow-up study or missed the data during the follow-up period. A total of 265 subjects with PACG were enrolled in the cohort study.  Figure 1 illustrates the flow chart of the cross-sectional and cohort studies.

### 2.3. Examination

The standardized ophthalmic examinations were conducted by a glaucoma specialist including measurement of anterior chamber angle (gonioscopy), IOP (Goldmann applanation tonometry, Haag-Streit, Bern, Switzerland), fundoscopy (TRC-NW200, Topcon Medical Systems, Oakland, New Jersey, USA), central corneal thickness (CCT), axial length (AL), and anterior chamber angle (ACD) (A-Scan Pachymeter, Ultrasonic, Exton, Pennsylvania, USA). The vertical cup-to-disc ratio (VCDR) was evaluated by two ophthalmologists and then averaged the values.

Medical examinations were conducted by speciality physicians from the Eye and ENT Hospital of Fudan University. All subjects were assessed height, weight, heart rate, blood pressure, electrocardiogram, radiograph, liver function, renal function, and infectious disease. The weight (kg) is divided by height into squares (m^2^) calculating the body mass index (BMI).

A fasting serum sample was collected via standard venipuncture from all subjects when they first time went to the Eye and ENT Hospital. All sample tubes were centrifuged at the speed of 3,000 rpm for 10 min, and all serum samples were completed the entire process within 3 hours. The levels of calcium and phosphorus were measured with the colorimetric method by calcium gen.2 (Ca2) and phosphate (Inorganic) ver.2 (PHOS2) (Roche Diagnostics GmbH, Mannheim, Germany). The levels of sodium, chlorine, and K^+^ were measured with Ion Selective Electrode Method (Roche COBAS C702, Roche Diagnostics GmbH, Mannheim, Germany). Internal controls were analyzed daily, and the variation coefficients were controlled between 2.1% and 3.8% with no significant value changes.

### 2.4. VF Analysis

The VF analysis was performed using the Octopus perimeter (G1 program, Haag-Streit, Bern, Switzerland), and the method was described previously [20, 22, 23]. After practicing at least 2 times, the PACG subjects took the VF examination, and a reliable and compatible VF mean deviation (MD) was recorded (false positive value < 15%, false − negative value < 15%, and reliability factor value < 20%). The VF examination was performed the first time when the PACG subject was recruited and every six months after that. Only the subjects having more than 5 reliable VF results and being followed up for more than 2 years were included in the statistical analysis. Meeting one or more following criteria can define the functional damage progression: (1) developing a new scotoma of ≥3 nonedge point worsening ≥5 dB, or one nonedge point worsening ≥10 dB; (2) a cluster of ≥3 nonedge points with ≥10 dB deterioration in a preexisting scotoma; (3) developing a new cluster of ≥3 nonedge points with 15° around a preexisting scotoma; (4) worsening of the global MD value by ≥2 dB per year [24]. One PACG eye which was randomly selected in subjects had bilateral PACG was observed in each subject during the entire follow-up period.

### 2.5. Statistical Analysis

Statistical analyses were performed by SPSS software (version 26; SPSS Inc., Chicago, Illinois, USA). Figures were created by GraphPad Prism (version 8.4; La Jolla, California, USA). A sample size calculation was undertaken to determine the study's recruitment sample size. We used an open-source calculator to calculate the minimal required sample size based on an odds ratio (OR) = 3, *α* = 0.05, *β* = 0.20, and a prevalence of PACG of about 10%. The obtained sample size for each group was 131 for the cross-sectional study. In the cohort study, assuming a VF endpoint in 45% of patients by the end of two years, the hazard ratio (HR) was 2. Fixing *α* = 0.05 and *β* = 0.20, the mean progress time was 12 months in the progression group, and 260 patients were needed for the analysis. The results were presented as mean ± standard deviation (SD) or percentage according to the data type. The normality of the data was tested by The Kolmogorov-Smirnoff test. Depending on the data type and distribution, one of the following methods was chosen to compare two groups: independent student *t*, Mann–Whitney *U*, Fisher's exact, or *χ*^2^ test. The association between the levels of serum ions and the risk of PACG was assessed by univariate (model A) and multivariate (model B and C) logistic regression analyses. The association between the serum ion levels and PACG progression was tested by univariate (model A) and multivariate (model B and C) COX regression analyses. Model B was adjusted age, and model C was adjusted age, BMI, hypertension (yes = 1, no = 0), and diabetes (yes = 1, no = 0). The results of logistical regression analyses were presented as OR (odds ratio) and corresponding 95% confidence intervals (CIs). The results of COX regression analyses were presented as HR (hazard ratio) and 95% CIs. In the cohort study, the PACG subjects were divided into K^+^ ≤ 4.3 and K^+^ > 4.3 based on the median K level. The MD and the changes of MD between K^+^ ≤ 4.3 and K^+^ > 4.3 groups were compared by the independent student *t*. Kaplan-Meier survival analysis was used to assess survival outcomes and the log-rank estimated the association between ion levels and PACG progression. A two-sided *P* value of < 0.05 was considered statistically significant in all analyses above.

## 3. Results

### 3.1. Demographic and Serum Ion Concentrations of the Study Subjects in the Cross-Sectional Study

The characteristics of 265 PACG and 166 normal subjects were summarized in Table 1. In PACG group, the average age was 62.71 years, and diabetes and hypertension rates were 7.55% and 20.38%, respectively, which showed no significant difference from the normal group. The PACG group was unbalanced between males (92, 34.72%) and females in accordance with the prevalence characteristics of PACG [25, 26]. The K^+^ levels (4.31 ± 0.39 mmol/L) were significantly higher in the PACG group than the normal group (4.16 ± 0.35 mmol/L, *P* < 0.001). The K^+^ levels in PACG subjects were significantly higher than in normal subjects in both males and females (*P* = 0.02 and *P* = 0.001, respectively).

### 3.2. Logistic Regression Analysis of the Association between Serum Ion Level and Risk of PACG

The logistic regression analysis was performed to assess the association between ion levels and the risk of PACG (Table 2). After adjusting for covariates in model C, the higher K^+^ levels were significantly associated with increased risk of PACG (OR = 2.94, 95%CI = 1.63 − 5.32, *P* < 0.001), also in the male (OR = 3.76, 95%CI = 1.20 − 11.78, *P* = 0.02) and the female (OR = 2.61, 95%CI = 1.30 − 5.32, *P* = 0.007).

### 3.3. Demographic and Serum Ion Concentrations of the Study Subjects in the Cohort Study

The baseline characteristics of 265 PACG subjects (160 without progression and 105 with progression) were summarized in Table 3. The baseline serum K^+^ level was significantly higher in PACG subjects with MD progression (4.41 ± 0.37 mmol/L) than in subjects without progression (4.25 ± 0.39 mmol/L, *P* = 0.002), which also showed significant differences in the male and female subgroups (*P* = 0.01 and *P* = 0.02, respectively).

### 3.4. MD of Subjects in Progression and No Progression Group

**Figure 2:**
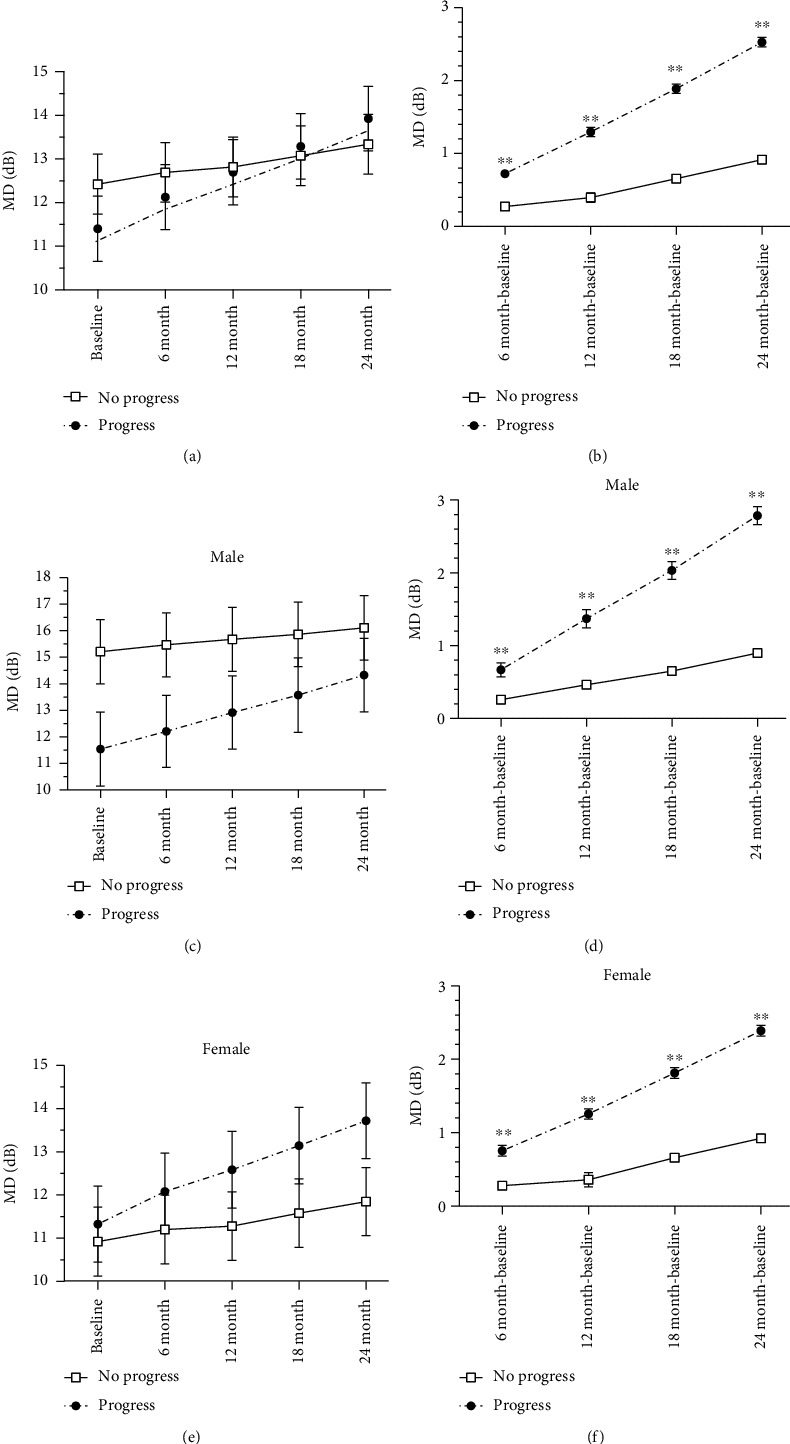
Comparison of the mean level of visual field mean deviation (MD) and mean change of visual field MD between progression and no progression groups in all PACG subjects (a, b), in males (c, d) and in females (e, f), ^∗^*P* < 0.05, ^∗∗^*P* < 0.001.

The mean MD level and the mean MD changes of PACG subjects in the progression and no progression group are shown in Figure 2. The mean MD levels have no significant difference between subjects with and without progression at baseline and the time point during the follow-up period (Figure 2(a)). However, the progression group had significantly higher mean changes of MD during the entire follow-up period (all *P* < 0.001) (Figure 2(b)), which also were found in males and females (all *P* < 0.001) (Figures 2(d) and 2(f)).

### 3.5. Cox Regression Analysis of the Association between Serum Ion Level and VF Progression in PACG

Table 4 shows the results of Cox proportional hazards regression analysis which assessed the association of baseline serum ion levels with the risk of PACG progression. In the fully adjusted model C, the higher baseline of K^+^ level has a significant association with PACG progression (HR = 2.07, 95%CI = 1.23 − 3.46, *P* = 0.006), which are also found in male and female subgroups (HR = 3.03, 95%CI = 1.15 − 7.96, *P* = 0.03; HR = 1.85, 95%CI = 1.00 − 3.41, *P* = 0.049, respectively).

### 3.6. Association between the Serum Potassium Level and Risk of PACG Progression

**Figure 3:**
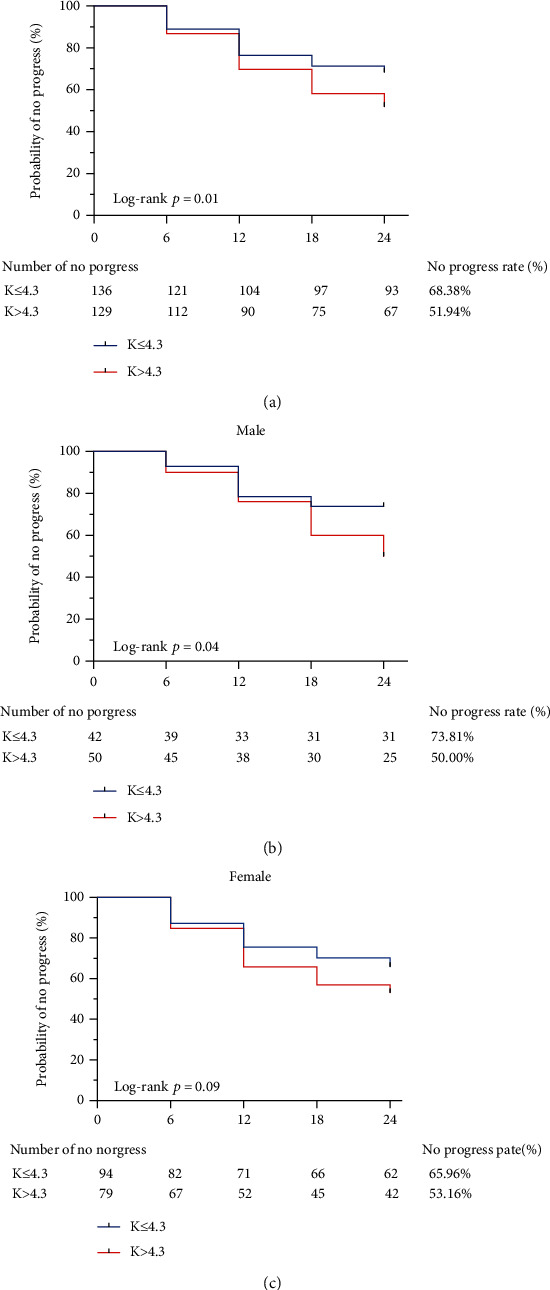
Kaplan-Meier curves for PACG patients divided by the median of potassium level. Plot of Kaplan-Meier depicts the probability of no progression in PACG patients in each group. The number of PACG patients without progression in each group was shown below five follow-up time points. Kaplan-Meier curves between lower and higher baseline levels of potassium in all PACG subjects (a), in male subjects (b), and in female subjects (c).

Based on the median baseline level of K, all PACG subjects were divided into the K^+^ ≤ 4.3 group and the K^+^ > 4.3 group. The Kaplan-Meier survival curves are shown in Figure 3. Compared with the K^+^ ≤ 4.3 group (68.38%), the K^+^ > 4.3 group has a significantly lower no-progress rate (51.94%; log-rank test *P* = 0.01). Figures 3(b) and 3(c) show similar trends in male (log-rank test, *P* = 0.04) and female (log-rank test, *P* = 0.09) PACG subjects.

## 4. Discussion

In this study, the association between serum ion levels (calcium, phosphorus, sodium, chlorine, and K^+^) and PACG was assessed. PACG subjects had a significantly higher level of K^+^ compared with normal subjects. The cohort study found that the higher serum level of K^+^ was significantly associated with VF progression in PACG subjects. Our findings suggested that increased K^+^ was a risk factor for the pathogenesis of PACG.

Proper K^+^ concentration in blood and extracellular fluid is critical to normal cellular function. The serum K^+^ level is maintained in normal concentration with a limited fluctuation [27]. During a 24 h period, the excretion of K^+^ has a circadian rhythm that plays a role in K^+^ regulation [28]. All serum samples were collected from PACG patients at fasting states in the morning. Thus, the serum K^+^ in each individual was considered to be representative. Furthermore, a rhesus monkey study reported that the concentrations of K^+^ were equal between aqueous humor and serum [29]. Ermolaev et al. reported that the concentration of K^+^ in aqueous humor was significantly higher than in serum, while K^+^ in aqueous humor and serum were equal in normal individuals [30]. The serum ion metabolism can import in retinal and brain and was associated with oxidative damage to glaucoma [31, 32]. Therefore, the serum level of K^+^ could imply the ocular level of K^+^ to a certain extent.

In the pathological process of PACG, the effect of K^+^ on VF defects remain unclear, and several feasible reasons might involve. The injury and death of RGCs are key pathogenic mechanisms of visual dysfunction [33]. Various ion channels are present at the RGCs, and frequent electrical activity might occur or change in the RGCs during the PACG onset and development [34, 35]. Among various ion channels, K^+^ channels are the most diverse and play key roles in modulating the electrical properties of RGCs, such as RGCs development, action potential, and repetitive firing regulation [36, 37]. Inward rectifier K^+^ channels increased could suppress the excitability of neurons [38], indicating that the increased serum K^+^ level could silence RGCs and cause the VF impairment. In addition, Müller cell, a glia cell spanning the entire retina, has the function of slowing glaucoma and regeneration of injured ocular cells [39]. Gao et al. reported that the changed expression and functions of K^+^ channels of Müller cell may contribute to glaucoma pathogenesis [40]. Therefore, it is possible that increased K^+^ level rapid VF deviation by injuring the function of Müller cell. Furthermore, ATP-dependent K^+^ channels were found in many mitochondrial or plasma membranes of ocular neuron cells [41, 42]. Dysregulation of these channels would injure retinal neuron cells, accompanied by oxidative stress and free radical accumulation.

Although a sudden and symptomatic elevation of IOP, which is caused by the iris covering the entire trabecular meshwork is the typical performance of PACG, difficulty in aqueous humor outflow through the trabecular meshwork and dysregulation of ocular blood flow also play a role in IOP elevation [43]. Stumpff et al. found that the efflux of K^+^ via maxi-K^+^ channels could result in trabecular meshwork relaxation [44]. A study detecting the trabecular meshwork cells derived from a normal subject and from a glaucoma patient found that the K^+^ current showed significantly different performance [45]. The altered K^+^ current might activate trabecular meshwork cell volume regulation, including cell shape changes and cell swelling, which obstructed aqueous humor outflow and resulted in increased IOP [46, 47].

Our study first reported that the higher serum level of K^+^ was a risk factor for PACG and VF progression. Limited data is available in the literature regarding the association of serum K+ levels with any type of glaucoma. Our laboratory explored the serum K+ level in primary open angle glaucoma (POAG). Unpublished data showed that the higher serum level of K+ is also a risk factor for onset and progression in POAG. However, it still has several limitations in the study. First, optical coherence tomography was not performed in this study. Therefore, the structural changes of the retina were not clear. Second, it is a single-center study. All participants enrolled in the cross-sectional study and cohort study were from the same hospital, which led to potential inclusion bias. Third, the concentration of K^+^ in aqueous humor was not detected in this study. Finally, operational treatments would lead to serum metabolite fluctuation and have an influence on visual field progression in the following period. Therefore, the PACG subjects enrolled in this study had no intraocular operation experience within the previous 2 months and during the entire follow-up period. However, the duration of PACG or the respective medications that the patients received before enrollment were not investigated, which might influence the progression of PACG. Prospective randomized controlled trials and basic experiments are needed in future.

## 5. Conclusion

In summary, we reported the increased serum level of K^+^ is significantly associated with PACG onset and progression, suggesting that K^+^ plays an important role in the pathogenesis and progression of PACG. The increased serum K^+^ might cause dysregulation of prooxidative and antioxidative processes in the ocular tissue of patients with glaucoma. Further prospective multicenter longitudinal studies and biological experiments are warranted to confirm our results and elucidate the underlying mechanisms.

## Figures and Tables

**Table 1:** Comparison of characteristics in primary angle close glaucoma and normal subjects.

Variable	Control group (*n* = 166)	PACG group (*n* = 265)	*t* value	*P* value
Age (year), mean ± SD	62.11 ± 7.54	62.71 ± 10.60	-0.56	0.57^a^
Sex (male, %)	49, 29.52%	92, 34.72%	1.25	0.26^b^
BMI (kg/m^2^), mean ± SD	22.68 ± 2.56	22.37 ± 3.49	0.85	0.40^a^
Diabetes, *n* (%)	8 (4.82%)	20 (7.55%)	1.25	0.26^b^
Hypertension, *n* (%)	38 (22.89%)	54 (20.38%)	0.38	0.54^b^
Topical glaucoma medications				
0-2		94 (35.47%)		
>2		171 (64.53%)		
IOP (mm Hg), mean ± SD		20.70 ± 16.71		
VCDR, mean ± SD		0.62 ± 0.28		
CCT (mm), mean ± SD		542.50 ± 41.62		
ACD (cm), mean ± SD		1.93 ± 0.49		
AL (cm), mean ± SD		22.48 ± 1.22		
MD (dB), mean ± SD		12.02 ± 8.29		
Calcium (mmol/L)	2.33 ± 0.09	2.33 ± 0.11	-0.50	0.62^a^
Male	2.32 ± 0.10	2.33 ± 0.12	-0.60	0.55^a^
Female	2.33 ± 0.09	2.34 ± 0.11	-0.20	0.84^a^
Phosphorus (mmol/L)	1.13 ± 0.16	1.16 ± 0.23	-1.51	0.13^a^
Male	1.03 ± 0.14	1.07 ± 0.16	-1.31	0.19^a^
Female	1.17 ± 0.15	1.21 ± 0.25	-1.65	0.10^a^
Potassium (mmol/L)	4.16 ± 0.35	4.31 ± 0.39	-4.28	<0.001^a^
Male	4.21 ± 0.40	4.36 ± 0.34	-2.38	0.02^a^
Female	4.14 ± 0.32	4.29 ± 0.41	-3.44	0.001^a^
Sodium (mmol/L)	141.79 ± 2.56	141.46 ± 2.39	1.33	0.18^a^
Male	141.59 ± 2.67	141.41 ± 2.25	0.42	0.68^a^
Female	141.87 ± 2.52	141.49 ± 2.47	1.28	0.20^a^
Chlorine (mmol/L)	102.77 ± 2.51	101.85 ± 2.91	3.36	0.001^a^
Male	102.12 ± 3.01	101.46 ± 2.93	1.27	0.21^a^
Female	103.04 ± 2.22	102.06 ± 2.89	3.10	0.002^a^

^a^Independent sample *t*-test. ^b^*x*^2^ test. ^c^Mann-Whitney *U* test.

**Table 2:** Logistic regression analysis to assess the value of baseline parameters associated with progression of primary angle close glaucoma.

	Model A		Model B		Model C	
	*P*	OR (95% CI)	*P*	OR (95% CI)	*P*	OR (95% CI)
Age	0.44	1.01 (0.99 to 1.03)				
Diabetes	0.51	1.33 (0.57 to 3.09)	0.63	1.23 (0.52 to 2.90)		
Hypertension	0.11	0.67 (0.42 to 1.09)	0.04	0.59 (0.36 to 0.97)		
BMI	0.54	0.98 (0.92 to 1.05)	0.44	0.97 (0.91 to 1.04)		
Calcium	0.62	1.62 (0.24 to 10.75)	0.58	1.71 (0.26 to 11.40)	0.65	1.61 (0.21 to 12.60)
Male	0.55	2.66 (0.11 to 63.73)	0.48	3.20 (0.13 to 79.71)	0.12	1.04 (0.99 to 1.08)
Female	0.85	1.26 (0.12 to 13.61)	0.84	1.29 (0.12 to 13.86)	0.72	0.62 (0.05 to 7.99)
Phosphorus	0.13	2.41 (0.76 to 7.60)	0.13	2.44 (0.77 to 7.71)	0.15	2.49 (0.71 to 8.69)
Male	0.19	4.97 (0.25 to 55.28)	0.16	5.83 (0.51 to 66.95)	0.03	25.30 (1.50 to 426.55)
Female	0.14	3.11 (0.69 to 14.10)	0.14	3.08 (0.68 to 13.95)	0.29	2.24 (0.50 to 9.98)
Potassium	<0.001	3.16 (1.83 to 5.46)	<0.001	3.15 (1.82 to 5.44)	<0.001	2.94 (1.63 to 5.32)
Male	0.02	3.25 (1.19 to 8.86)	0.02	3.21 (1.18 to 8.77)	0.02	3.76 (1.20 to 11.78)
Female	0.001	3.04 (1.58 to 5.85)	0.001	3.03 (1.57 to 5.84)	0.007	2.61 (1.30 to 5.22)
Sodium	0.18	0.95 (0.87 to 1.03)	0.17	0.95 (0.87 to 1.03)	0.11	0.93 (0.85 to 1.02)
Male	0.67	0.97 (0.84 to 1.12)	0.67	0.97 (0.84 to 1.12)	0.39	0.93 (0.79 to 1.10)
Female	0.20	0.94 (0.85 to 1.03)	0.19	0.94 (0.85 to 1.03)	0.18	0.93 (0.84 to 1.03)
Chlorine	0.001	0.88 (0.82 to 0.95)	0.001	0.88 (0.82 to 0.95)	<0.001	0.86 (0.79 to 0.93)
Male	0.21	0.93 (0.82 to 1.04)	0.20	0.92 (0.82 to 1.04)	0.04	0.86 (0.74 to 0.99)
Female	0.003	0.86 (0.78 to 0.95)	0.003	0.86 (0.78 to 0.95)	0.003	0.85 (0.77 to 0.95)

BMI: body mass index. Model A is not adjusted. Model B is adjusted for age. Model C was adjusted for age, BMI, diabetes (yes = 1, no = 0), and hypertension (yes = 1, no = 0).

**Table 3:** Comparison of characteristics in primary angle close glaucoma subject.

Variable	No progression (*n* = 160)	Progression (*n* = 105)	*t* value	*P* value
Age (year), mean ± SD	62.17 ± 10.92	63.38 ± 10.06	-0.91	0.36^a^
BMI (kg/m^2^), mean ± SD	22.76 ± 3.27	22.59 ± 3.34	0.41	0.68^a^
SBP (mm Hg), mean ± SD	72.14 ± 9.77	71.16 ± 10.20	0.78	0.44^a^
DBP (mm Hg), mean ± SD	128.59 ± 15.87	127.13 ± 17.60	0.70	0.48^a^
Diabetes, *n* (%)	11 (6.88%)	9 (8.57%)	0.26	0.61^b^
Hypertension, *n* (%)	33 (20.63%)	21 (20.00%)	0.02	0.90^b^
Topical glaucoma medications				
0-2	56 (35%)	38 (36.19%)	0.04	0.84^b^
>2	104 (65%)	67 (63.81%)		
IOP (mm Hg), mean ± SD	19.16 ± 9.57	23.04 ± 23.65	-1.60	0.06^a^
VCDR, mean ± SD	0.64 ± 0.30	0.58 ± 0.23	1.84	0.07^a^
CCT (mm), mean ± SD	545.21 ± 40.56	538.22 ± 43.11	1.30	0.19^a^
ACD (cm), mean ± SD	1.92 ± 0.53	1.94 ± 0.44	-0.26	0.80^a^
AL (cm), mean ± SD	22.45 ± 1.33	22.54 ± 1.06	-0.59	0.56^a^
MD (dB), mean ± SD	12.42 ± 8.69	11.40 ± 7.64	1.01	0.32^a^
Calcium (mmol/L)	2.32 ± 0.11	2.35 ± 0.11	-1.87	0.06^a^
Male	2.31 ± 0.12	2.37 ± 0.10	-2.36	0.02^a^
Female	2.33 ± 0.11	2.34 ± 0.11	-0.56	0.58^a^
Phosphorus (mmol/L)	1.15 ± 0.16	1.18 ± 0.31	-0.81	0.42^a^
Male	1.08 ± 0.17	1.05 ± 0.14	0.84	0.40^a^
Female	1.19 ± 0.15	1.24 ± 0.36	-1.26	0.21^a^
Potassium (mmol/L)	4.25 ± 0.39	4.41 ± 0.37	-3.17	0.002^a^
Male	4.29 ± 0.34	4.47 ± 0.31	-2.55	0.01^a^
Female	4.24 ± 0.41	4.37 ± 0.40	-2.18	0.03^a^
Sodium (mmol/L)	141.45 ± 2.54	141.49 ± 2.16	-0.12	0.91^a^
Male	141.55 ± 2.39	141.19 ± 2.03	0.75	0.46^a^
Female	141.39 ± 2.63	141.64 ± 2.22	-0.63	0.53^a^
Chlorine (mmol/L)	101.78 ± 3.24	101.96 ± 2.34	-0.49	0.62^a^
Male	101.64 ± 3.34	101.17 ± 2.16	0.76	0.45^a^
Female	101.86 ± 3.19	102.38 ± 2.35	-1.24	0.22^a^

^a^Independent sample *t*-test. ^b^*x*^2^ test. ^c^Mann-Whitney *U* test.

**Table 4:** Cox proportional hazards regression analysis to assess the value of baseline parameters associated with progression of primary angle close glaucoma.

	Model A		Model B		Model C	
	*P*	HR (95% CI)	*P*	HR (95% CI)	*P*	HR (95% CI)
Age	0.34	1.01 (0.99 to 1.03)				
Diabetes	0.71	1.14 (0.58 to 2.26)	0.84	1.08 (0.54 to 2.16)		
Hypertension	0.90	0.97 (0.60 to 1.57)	0.71	0.91 (0.56 to 1.49)		
BMI	0.67	0.99 (0.93 to 1.05)	0.61	0.98 (0.93 to 1.05)		
Calcium	0.11	3.87 (0.72 to 20.77)	0.08	4.89 (0.85 to 28.03)	0.08	4.78 (0.83 to 27.55)
Male	0.03	16.38 (1.25 to 214.59)	0.03	22.46 (1.33 to 378.59)	0.02	29.41 (1.56 to 555.48)
Female	0.71	1.51 (0.17 to 13.39)	0.61	1.02 (0.99 to 1.04)	0.68	1.62 (0.17 to 15.68)
Phosphorus	0.54	1.24 (0.62 to 2.47)	0.51	1.25 (0.64 to 2.48)	0.52	1.26 (0.63 to 2.54)
Male	0.40	0.39 (0.04 to 3.47)	0.35	0.34 (0.03 to 3.34)	0.60	0.52 (0.05 to 6.02)
Female	0.34	1.39 (0.71 to 2.72)	0.36	1.37 (0.69 to 2.71)	0.26	1.52 (0.73 to 3.16)
Potassium	0.005	2.07 (1.24 to 3.44)	0.005	2.07 (1.24 to 3.44)	0.006	2.07 (1.23 to 3.46)
Male	0.03	2.79 (1.08 to 7.22)	0.04	2.79 (1.08 to 7.21)	0.03	3.03 (1.15 to 7.96)
Female	0.045	1.84 (1.02 to 3.34)	0.050	1.82 (1.00 to 3.31)	0.049	1.85 (1.00 to 3.41)
Sodium	0.91	1.01 (0.93 to 1.09)	0.92	1.00 (0.93 to 1.09)	0.95	1.00 (0.93 to 1.09)
Male	0.52	0.95 (0.83 to 1.10)	0.52	0.95 (0.83 to 1.10)	0.42	0.94 (0.80 to 1.10)
Female	0.58	1.03 (0.93 to 1.13)	0.57	1.03 (0.93 to 1.13)	0.61	1.03 (0.93 to 1.14)
Chlorine	0.65	1.02 (0.95 to 1.08)	0.32	1.01 (0.99 to 1.03)	0.59	1.02 (0.95 to 1.09)
Male	0.45	0.96 (0.87 to 1.07)	0.45	0.96 (0.87 to 1.07)	0.20	0.93 (0.82 to 1.04)
Female	0.28	1.05 (0.96 to 1.14)	0.22	1.06 (0.97 to 1.15)	0.18	1.06 (0.97 to 1.16)

BMI: body mass index. Model A is not adjusted. Model B adjusted for age. Model C was adjusted for age, BMI, diabetes (yes = 1, no = 0), and hypertension (yes = 1, no = 0).

## Data Availability

The datasets used and/or analyzed during the current study are available from the corresponding author on reasonable request.
